# Postural Stability Evaluation of Patients Undergoing Vestibular Schwannoma Microsurgery Employing the Inertial Measurement Unit

**DOI:** 10.1155/2018/2818063

**Published:** 2018-04-18

**Authors:** Patrik Kutilek, Zdenek Svoboda, Ondrej Cakrt, Karel Hana, Martin Chovanec

**Affiliations:** ^1^Faculty of Biomedical Engineering, Czech Technical University in Prague, Sitna Sq 3105, Kladno, Czech Republic; ^2^Faculty of Physical Culture, Palacký University, Tr. Miru 115, Olomouc, Czech Republic; ^3^Motol University Hospital, Charles University, V Uvalu 84, Prague, Czech Republic; ^4^Third Faculty of Medicine, Charles University in Prague, Ruská 2411/87, Prague, Czech Republic

## Abstract

The article focuses on a noninvasive method and system of quantifying postural stability of patients undergoing vestibular schwannoma microsurgery. Recent alternatives quantifying human postural stability are rather limited. The major drawback is that the posturography system can evaluate only two physical quantities of body movement and can be measured only on a transverse plane. A complex movement pattern can be, however, described more precisely while using three physical quantities of 3-D movement. This is the reason why an inertial measurement unit (Xsens MTx unit), through which we obtained 3-D data (three Euler angles or three orthogonal accelerations), was placed on the patient's trunk. Having employed this novel method based on the volume of irregular polyhedron of 3-D body movement during quiet standing, it was possible to evaluate postural stability. To identify and evaluate pathological balance control of patients undergoing vestibular schwannoma microsurgery, it was necessary to calculate the volume polyhedron using the 3-D Leibniz method and to plot three variables against each other. For the needs of this study, measurements and statistical analysis were made on nine patients. The results obtained by the inertial measurement unit showed no evidence of improvement in postural stability shortly after surgery (4 days). The results were consistent with the results obtained by the posturography system. The evaluated translation variables (acceleration) and rotary variables (angles) measured by the inertial measurement unit correlate strongly with the results of the posturography system. The proposed method and application of the inertial measurement unit for the purpose of measuring patients with vestibular schwannoma appear to be suitable for medical practice. Moreover, the inertial measurement unit is portable and, when compared to other traditional posturography systems, economically affordable. Inertial measurement units can alternatively be implemented in mobile phones or watches.

## 1. Introduction

Sensory nervous system disorders adversely affect postural stability of patients [1]. Patients with vestibular schwannoma (VS), a benign tumor originating from Schwann cells of vestibular part XIII of the cranial nerve, encounter—in addition to other problems—balance and vertigo disorders [1–3]. The slow VS growth leads to gradual vestibular dysfunction and deteriorates postural stability of stance, which is partially compensated for by the central compensatory mechanism [4].

In clinical practice, stabilometric platforms are employed for the evaluation of patients' postural stability. These platforms monitor the movement in the centre of pressure (CoP); subsequently, the motion is assessed by means of quantitative CoP indicators [1, 5]. Stabilometric platforms are, however, expensive when compared to motion sensors based on gyroscopes or accelerometers (also used in modern phones and watches).

Stabilometric platforms do not allow for the study of individual body segments' movement. Their other drawback is that they can monitor the movement of a body only in the transverse and not the other anatomical planes (frontal and sagittal). For these reasons, in patients with instability disorders, inertial measurement units (IMU) are increasingly being used for the accurate measurement of movement of specific body segments [6]. IMU enable the measurement of translational and angular movements in all three perpendicular planes of the three-dimensional (3-D) space, that is, three angles and three accelerations.

In clinical practice, the IMU commonly used for quantification of body segment movement are only for measurement and evaluation of one or two measured quantities, usually sway and angles [7]. For these reasons, the introduction of IMU and newly developed methods assessing body segment movement in people with postural stability disorders has great potential in clinical practice. In clinical practice, the 3-D IMU have not been used for the assessment of patients with VS yet and neither the practical applicability of these units for VS treatment has been proven. The first objective of the presented work is to demonstrate how IMU can be used for the assessment of postural stability in patients with VS and how this assessment compares with one conducted by stabilometric platforms, which are currently used in clinical practice [1], and, furthermore, to ascertain whether IMU results are consistent with the results achieved by using the stabilometric platforms.

The second objective, which, from a technical point of view, is equally important, is to propose and present a suitable method for quantitative assessment of the 3-D motion measured by IMU for postural stability in stance. Traditional methods used for quantitative assessments of postural stability in stance are usually based on the processing of two-dimensional (2-D) data from stabilometric platforms [8].

The selected assessment method of 2-D data will be adjusted to assess 3-D data, and the results obtained by this modified method will be compared with those obtained from the stabilometric platform. The reason for using a complete set of 3-D data, assessing postural stability of stance, is that using just two variables in 3-D motion may cause a loss of information about the third component of motion of a particular segment in space.

Using a quantitative assessment of the 3-D data, information on the overall movement of a particular body segment in the 3-D space can be obtained. For further research, a segment on a patient's trunk was found as the most suitable to attach the IMU system. A similar application measuring angles of movement (MoCap) has been already successfully used in assessing postural stability in stance [9].

The final aim of this work, in terms of clinical application, is to demonstrate the difference in a patient's stability while performing different stance tasks both pre- and postsurgery. The outcome of this test is to be a statement, whether there is any change in postural stability of patients shortly after the surgery [10]. Such measurements are commonly conducted in a patient's upright position under different visual conditions (with open and closed eyes) and on different surfaces (firm versus foam surface) [1]. This measurement will allow for comparison with the more expensive stabilometric platform. In addition, this method of examination will also be used to examine postural stability of patients with an IMU.

## 2. Materials and Methods

### 2.1. Participants

Nine patients undergoing VS microsurgery were involved in the study. The surgery was performed in Motol University Hospital in the Department of Otolaryngology and Head and Neck Surgery of the 1st Faculty of Medicine, Charles University, in Prague. The subjects for measurement were randomly selected from the first half of 2014 to the first half of 2015. The patients, comprising of four men and five women averaging 46.7 (SD 11.9) years of age, were subjected to the measurement twice: before surgery and then 4 days after surgery.

Audiometric (pure-tone audiometry, speech audiometry, stapedial reflex, otoacoustic emissions, and brainstem auditory-evoked potentials) and neurootologic tests (clinical testing, electronystagmography, spontaneous nystagmus, gaze directional test, saccades, smooth pursuit, caloric test and head impulse and head shaking test, subjective visual vertical, and posturography) were taken along with magnetic resonance imaging in all the patients.

All nine patients were operated on by the same team of surgeons, using the retrosigmoid-transmeatal approach in the supine position. The surgeons used microsurgical endoscopy and techniques with intraoperative neuromonitoring. In all nine cases, the tumors were removed. The section of both vestibular parts of the 8th cranial nerve was performed even in the cases where continuity could be preserved.

The study was performed in accordance with the Declaration of Helsinki. The study protocol was approved by the local Ethical Committee of Motol University Hospital, and informed consent was obtained from all the subjects involved.

### 2.2. Test Procedure and Measurement Equipment

**Figure 1:**
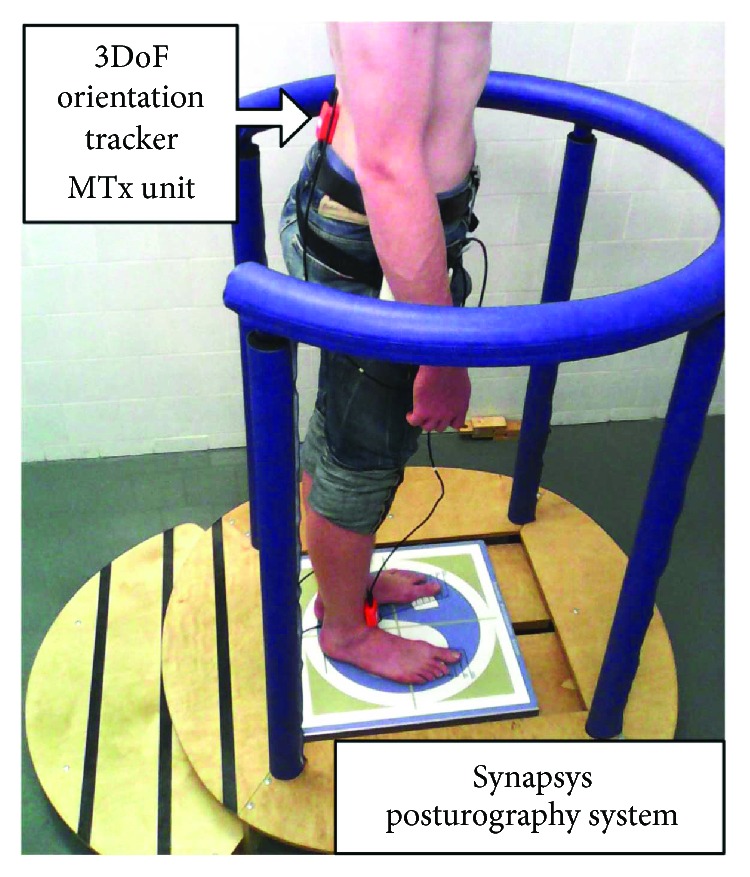
The MTx unit employed to measure angles and accelerations of the trunk and the Synapsys posturography system used to measure the CoP displacements.

To measure trunk movements, specifically shift and sway, we used the Xbus Master (Xsens Technologies B.V.), a lightweight (330 g) and portable device using MTx units for orientation and acceleration measurement of body segments (see Figure 1). The MTx unit has an embedded accelerometer and gyroscope. It is considered an accurate IMU, measuring drift-free 3-D orientation and 3-D acceleration.

The MTx unit was calibrated before each clinical examination. The MTx unit was set up in the following ways: one axis of the MTx's coordinate system was parallel to the anterior-posterior axis, that is, the symmetry axis of the fixed stationary platform of the Synapsys posturography system, on which the participants stood. The other two axes were perpendicular to the anterior-posterior axis (i.e., the symmetry axis of the platform) respecting the Earth's gravitational direction, that is, the superior-inferior axis was colinear with the direction of gravity. After calibration, the MTx unit was placed on the patient's trunk according to [9, 11, 12], at the level of the lower back (lumbar 2-3, see Figure 1).

The data, that is, the three Euler angles (roll (*Φ*), yaw (Ψ), and pitch (Θ)) [13, 14] and three orthogonal accelerations (a_Sx_, a_Sy_, and a_Sz_), in the accelerometer coordinate system [15] were measured by the MTx unit placed on the patient's trunk (Pts) and healthy subjects (control group (CG)) while they were standing still on a fixed stationary platform of the Synapsys posturography system. Conventions for the Euler angles are described in [13, 16, 17]. The three accelerations measured by the MTx accelerometer unit were described previously [18, 19].

The data, obtained from the IMU, were compared with the data obtained by the traditional method based on CoP measurement. The values of centre of pressure (CoP) displacement (i.e., postural sway) were measured by a posturography system, the Synapsys posturography system (Synapsys Inc.).

Body sway was measured by the Xsens system and Synapsys posturography system during a still stance on a firm surface (FiS) and a soft foam surface (FoS) with eyes open (EO) and eyes closed (EC) [20]. The subject's bare feet were positioned next to each other, splayed at a 30° angle, arms always in natural hanging position. The tasks included standing on both feet for at least 60 seconds [21]. The two systems recorded body activities simultaneously. Time synchronization of the measured data (i.e., data from both systems) was secured by control and data processing on the same computer. The measurements usually lasted a few seconds longer, and the initial data period was shortened, so that all datasets had a record length of 60 seconds. The data was recorded at the sample frequency of 100 Hz.

### 2.3. Method of Data Processing

The three Euler angles and three accelerations in the accelerometer coordinate system were used to calculate the accelerations in the global reference system and then in the anatomical coordinate frame. The calculation was based on the rotational matrices. The rotation matrices rotate an acceleration vector ${\vec{a}}_{S}={\left(a_{Sx}a_{Sy}a_{Sz}\right)}^{T}$ in the sensor coordinate system to the anatomical coordinate frame, where the matrices are interpreted in terms of Euler angles [22]. The calculated acceleration vector ${\vec{a}}_{A}={\left(\begin{array}{ccc} a_{AP} & a_{ML} & a_{SI} \end{array}\right)}^{T}$ represents the superior-inferior acceleration (**a**_SI_), mediolateral acceleration (**a**_ML_), and anterior-posterior acceleration (**a**_AP_). The acceleration vectors, or in other words time-dependent data (**a**_SI_, **a**_ML_, and **a**_AP_), are plotted as a 3-D plot. The set of points is obtained by plotting the accelerations against each other. The time of measurement, that is, record length of the dataset (60 s) and the sample frequency (100 Hz), determines the number of points in the set.

The novel method for identification of pathologies affecting balance control is based on the mathematical tools for static posturography [8, 23]. The distribution of the measured data can be modelled by a method which refers to the sector formula of Leibniz [8]. The graphical result of the method is a polygon envelope containing the measured data. The 2-D Leibniz method has already been used in clinical practice to study postural balance problems [8], but the concept of the polyhedron envelope has not been used in clinical practice for the purpose of studying the postural balance problems by three accelerations or three angles. Here, a method based on the description of distribution of the measured data (i.e., **a**_SI_, **a**_ML_, and **a**_AP_ and/or *Φ*, Ψ, and Θ) by polyhedron envelope has been applied.

**Figure 2:**
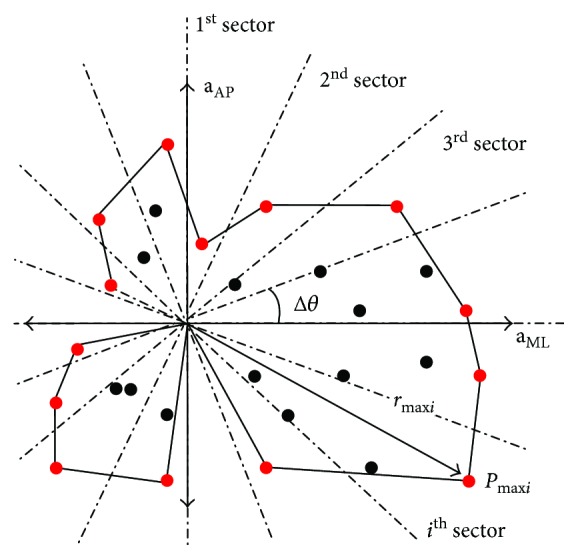
Polygon area calculation via the 2-D Leibniz method and by plotting mediolateral (ML) and anterior-posterior (AP) accelerations versus each other.

In more detail, the original Leibniz method proposes an evaluation of stability by calculating the area bounded by the 2-D data points located in predefined zones relative to the median of the 2-D data. The 2-D data, which is obtained from a measurement system, is able to register the variation of **a**_AP_ and a_ML_ (or *Φ* and Ψ). In the case of the 2-D space, the area is determined by the most distant points *P*_max_ of data subsets to the median of the data, that is, the points with the maximum radius value (*r*_max_) (see Figure 2). The data subsets belong to the sectors of the 2-D space (see Figure 2). For this purpose, the 2-D plot is divided in equal angles from the centre (i.e., median of the data) ranging from 0° to 360°. The data subsets are defined by a predefined differential angle (Δ*θ*) and axis with infinite radius (see Figure 2) [8].

**Figure 3:**
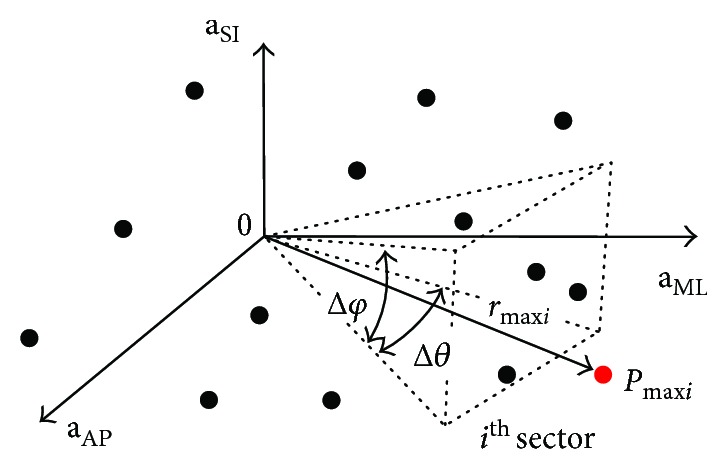
Divide spherical space into subsets of the points obtained by plotting superior-inferior (SI), mediolateral (ML), and anterior-posterior (AP) accelerations versus each other.

Such a method can be modified for stability evaluation by calculating the volume bounded by the 3-D data points located in the predefined zones relative to the median of 3-D data (**a**_AP_, **a**_ML_, and **a**_SI_ or *Φ*, Ψ, and Θ). Regarding the 3-D space, the volume of irregular polyhedron is determined by calculating the furthest points from the median found in specific sectors (see Figure 3). The algorithm designed to calculate the polyhedron is described in Table 1. This method is based on the conversion of the Cartesian coordinates to spherical coordinates, while entering the radial distance (*r*), azimuthal angle (*φ*), and elevation angle (*θ*), and on the division of spherical space into *i* subsets belonging to *i* sectors of the 3-D space defined by predefined Δ*φ* and Δ*θ*. The total number of sectors is given by the predefined size of Δ*φ* and Δ*θ*. Due to previous experiments considering the computational complexity of the algorithm, Δ*φ* and Δ*θ* were 10 degrees.

**Figure 4:**
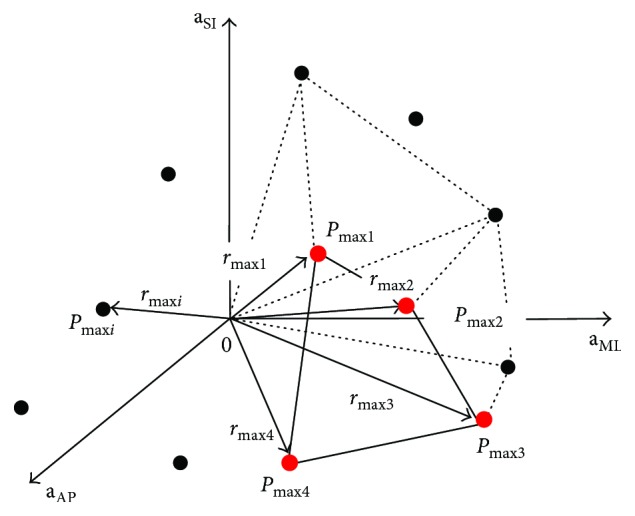
Volume polyhedron calculation by the 3-D Leibniz method and plotting superior-inferior (SI), mediolateral (ML), and anterior-posterior (AP) accelerations versus each other.

The method used to calculate the volume of each elemental pyramid is based on a calculation of the convex polyhedron volume [23]. In mathematics, the convex hull of a set of points (SP) in the Euclidean space is the smallest convex set that contains SP [24, 25]. The SP contains one point, that is, vertex of pyramid, at the origin of reference frame *P*_0_(0, 0, 0) and four points *P*_max_(*x*, *y*, *z*), vertices of pyramid with maximum radius values *r*_max_ among all points in four subsets belonging to four adjacent sectors (see Figure 4). A custom-designed MatLab program based on the functions of the MatLab software (MatLab R2010b, Mathworks Inc., Natick, MA, USA) was used to divide the 3-D space into sectors, to identify the points with maximum radius values and to calculate the convex polyhedron volumes.

**Figure 5:**
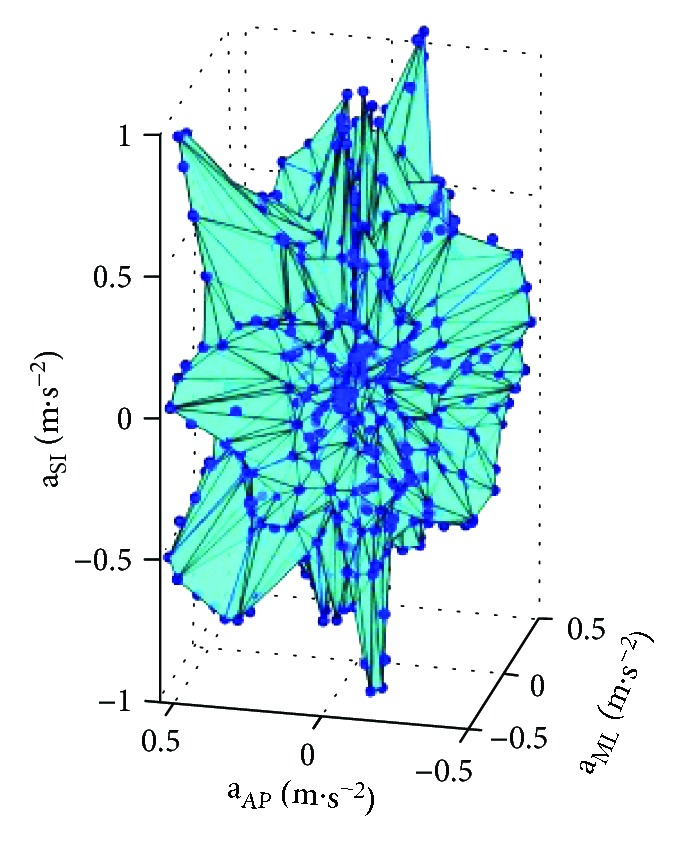
An example of a polyhedron obtained by plotting superior-inferior (SI), mediolateral (ML), and anterior-posterior (AP) accelerations versus each other.

For a convex polyhedron computation in MatLab, Delaunay triangulation [26, 27] was used, (see Figure 5). Since no other method for calculating the convex polyhedron volume [28, 29] is known, equations for the calculation of volume of any polyhedron may be used. The method of the calculation of convex polyhedron volume is described in detail in [30]. The final volume of the 3-D polyhedron (TVP) is determined as the total of all the pyramid volumes, that is, elemental polyhedron volumes. Since the volume corresponds with the volume of 3-D polyhedron obtained by plotting **a**_SI_, **a**_ML_, and **a**_AP_ against each other (i.e., total volume of the polyhedron of accelerations (TVPA)), or by plotting *Φ*, Ψ, and Θ against each other (i.e., total volume of the polyhedron of angles in degrees (TVPD)), the physical unit of the volume is represented by m^3^·s^−6^ or deg^3^. It is also necessary to mention that the MTx unit also records gravitational acceleration which does not have to be subtracted. The calculation of the polyhedron volume exploits only changes in the accelerations, and the gravitational acceleration is constant and perpendicular to the horizontal plane of the Earth's surface. In spite of this, gravitational acceleration was also subtracted in the MatLab software. To compare the data obtained by the posturography system with data obtained by the IMU, the area of the 95% confidence ellipse (ACE) and 2-dimensional path length (PL) of CoP excursions was used. The Synapsys posturography system directly calculated the areas and lengths, so the measured data did not have to be converted. The physical unit of the area is one mm^2^ whereas that of the length is one mm [8].

### 2.4. Statistical Analysis

After calculating the TVP, ACE, and PL of each patient pre- and postsurgery (while standing on a FiS and FoS with EO and EC), the Jarque–Bera test was used to identify a normal distribution of calculated characteristics [31]. The median (Mdn), minimum (Min), maximum (Max), the first quartile (Q1), and the third quartile (Q3) of the TVP, ACE, and PL were used to compare the results. The Wilcoxon signed rank test assessed the significance of the differences between the results of measurements. The significance level was set at *p* < 0.05. The Spearman's rank correlation coefficient between the TVP, ACE, and PL was calculated to identify the differences between the data from IMU and the data from the posturography system. In addition, optimal sizes, considering differences between the two groups of data, were calculated in accordance with [32, 33]. The statistical analysis was performed by MatLab software.

## 3. Results

**Figure 6:**
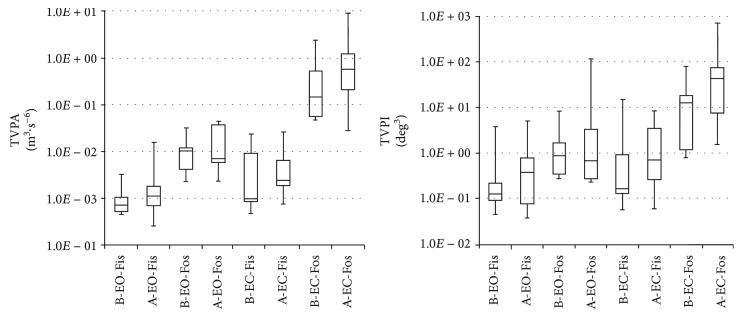
Comparison of the data measured by IMU; TVPA: total volume of the polyhedron of the accelerations; TVPD: total volume of polyhedron of the angles; EO: eyes open; EC: eyes closed; FiS: firm surface; FoS: foam surface; B: presurgery; A: postsurgery.

**Figure 7:**
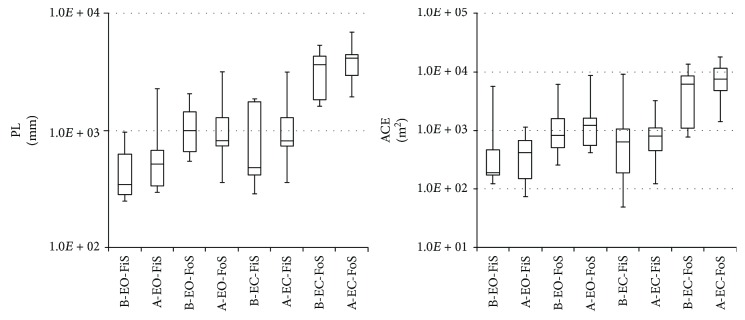
Comparison of the data measured by posturography system; ACE: area of the confidence ellipse; PL: path length of CoP; EO: eyes open; EC: eyes closed; FiS: firm surface; FoS: foam surface; B: presurgery; A: postsurgery.

The statistical data illustrate the differences in stance trials pre- and postsurgery (Tables 2–5). The following plots (Figures 6 and 7) display the Min, Max, Mdn, Q1, and Q3 for the calculated TVPAs, TVPDs, ACEs, and PLs. Since some calculated values were not distributed as expected, the Wilcoxon test was used to compare and analyse the calculated data sets.

### 3.1. Comparing the Quiet Stance Trials

In almost all the cases, statistically significant differences were found in the data comparing stance trials (see Table 6). In the case of the Pts with EO and EC standing on the FiS and FoS, the measured data shows a significant increase of the median of the TVPAs, TVPDs, ACEs, or PLs after the eyes closed and standing on the FoS (Figures 6 and 7).

### 3.2. Correlation between the Data from IMU and the Stabilometric Platform

In the case of patients with EO standing on the FiS and FoS, the Spearman's rank correlation coefficient indicates significant correlation between the TVPA or TVPD and ACE or PL. In the both cases, that is, pre- and postmicrosurgery, the correlations are moderate or slightly higher. In most cases, the patient examinations show strong or very strong positive correlations between the data measured by IMU and that measured by the posturographic platform (see Tables 7 and 8).

### 3.3. Comparing Patients Pre- and Postsurgery

No significant differences were found when comparing Pts pre- and postmicrosurgery with EO standing on FiS or FoS. A significant difference was observed only when comparing Pts pre- and postmicrosurgery with EC standing on FoS. A significant difference was observed by TVPD and PL, (see Table 9).

The median of the TVPD in Pts postmicrosurgery is 3.4 times higher than the median of the TVPD in Pts presurgery. The median of the PL in Pts postsurgery is 1.2 times higher than the median of the PL in Pts presurgery. In all cases, the comparison of data showed that the effect sizes were moderate to large, that is, calculated values were higher than 0.4.

## 4. Discussion

The aim of the study was to demonstrate the applicability of an IMU for assessing postural stability of patients with VS. In almost all cases, changes in stance conditions, that is, EO versus EC and FiS versus FoS, resulted in a statistically significant change of TVPA, TVPD, ACE, and PL values.

This study concludes that complicated stance tasks performed when either a mechanoreceptor or visual perception is reduced have a significant impact on trunk movements. The findings are consistent with those obtained when healthy subjects standing with EC were measured on FoS [34, 35]. The results also show that the measurement of postural stability under different conditions by means an IMU positioned on the patient's trunk leads to the same results as those measured using a more expensive posturography system. The IMU, employing TVPA and TVPD indicators, can be used in clinical trials for assessing postural stability rather than the traditional posturography system.

It was also found that there was a strong correlation between the recorded results from the posturography system achieved by the quantitative methods and those from the IMU. In most cases, they carried strong positive correlation characters. Conversely, no significant difference in terms of correlation was found between the results achieved by the TVPA method and those by TVPD with ACE and PL. Both methods correlate with ACE and PL similarly, data obtained by evaluation of accelerations, that is, from translational 3-D motion, reach the same conclusions as the data obtained by angle evaluation, that is, angular 3-D motion. The reason is that the large movements of the trunk, which improves the stability of the patient's body, have a great impact on changing the centre of mass (CoM) of the whole body which corresponds to the position of the CoP [36]. It is assumed that translational and angular movements of the trunk are related; as soon as the trunk tilts, the sensors tilt in the same direction. Comparison of postural stability of VS patients before and shortly after surgery revealed that in a great majority of cases, no statistically significant changes in postural stability were recorded pre- and postsurgery. All the indicators, however, refer to a significantly decreased *p* value when comparing the stance of patients pre- and post- EC surgery to FoS. In some cases of angular 3-D trunk movement - a TVPD indicator, and path length of CoP discovered that there was a slight but statistically significant deterioration in stability (i.e., *p* < 0.05).

The reason for the deterioration above may be that patient measurements were performed shortly after surgery, when they still did not undergo complete postoperative recovery. When measuring of postural stability was performed 8 days postsurgery (see [10]), similar findings were recorded and presented.

Based on the results, it can be said that the position CoP of the whole body, which is traditionally subjected to assessments in patients with VS, is significantly affected by the position of their trunk. The results also show that in clinical practice, it is more suitable to perform the examination of subjects in a standing position with the EC placed on FoS since the postural control deficits can be identified more accurately.

As we have mentioned above, the measurement methodology is commonly used to measure postural stability by means of stabilometric platforms in clinical practice. Validation of the application of the inertial measurement unit was performed by comparative measurement using the stabilometric platform (see Tables 7 and 8). Comparison of data was performed repeatedly before and after operation of patients and under different conditions (standing with EC and EO on FoS and FiS) of measurement.

Nevertheless, there are some limitations to this research study. The most important is that the sample size of the subjects was too small and may not have been representative enough of the larger population. However, nine patients proved to be sufficient for the preliminary research which managed to test the basic attributes of the method proposed for further studies of postural stability. The size of the sample group can be compared to one used in similar research focused on studying the vestibular system [37].

It was also deemed unnecessary to compare patients of different ages, since the results demonstrate that the parameters of body sway of healthy subjects within the age range of 20 to 60 years vary only insignificantly [38, 39]. Aoki et al. [38] found that only insignificant differences were recognized in the age group of 10- to 60-year-old subjects in CoP sway parameters (i.e., Romberg quotients). Moreover, the detailed analysis of age-related increases of CoP parameters by the polynomial type of regression showed that the gradual increase of body sway, that is, significant degradation of stability characterized by increase of CoP oscillations started after the age of 60 [39]. Another limitation in this research was that only one single measurement of each subject was taken, although subjects with impaired postural stability are usually measured only once in general or only data from trials with the most complete and longest record are used for further analysis [40].

## 5. Conclusions

The research conducted and this follow-up study on postural instability in patients with VS using an IMU placed on the patient's trunk and the Leibniz method show that the method presented is suitable for the identification of postural balance problems. This technique described allows for the study of three measured variables (three angles and three accelerations) of 3-D body movement [41, 42]. Adopting this approach overcomes the greatest limitation of traditional methods relying on just two variables, each in one of the two human body axes of the transverse level (as used in CoP research) [41, 42]. Although this new technique has never been used for the study of patients with VS, its application can be found in other fields, for data analysis obtained by an IMU in cell phones or electronic watches. This new technological application can lead to greater use of the proposed methods in small health clinics or even at home [41, 42].

Reviewing the clinical findings, the measurement results are in line with the results of previous studies used for evaluation of stability on platforms. Although the postural stability of patients did not improve four days post operation, it is expected to improve after a complete recovery. It is therefore essential for new or subsequent research of patients with VS to focus on IMU testing in postural stability assessment over the long-term course of the recovery process.

## Figures and Tables

**Table 1:** Algorithm for calculation of the total volume of 3-D polyhedron.

(1) Calculate the median of the distribution of *x*, *y*, and *z* variables, where *x* is **a**_AP_ (or *Φ*), *y* is **a**_ML_ (or Ψ), and *z* is **a**_SI_ (or Θ).
(2) Set the origin of the frame of reference to the median of the distribution.
(3) Convert the Cartesian coordinates of each data point *P*(*x*, *y*, *z*) of the three variables measured at the same specific time into spherical coordinates (*P*(*r*, *φ*, *θ*)).
(4) Divide the data into subsets belonging to sectors of the 3-D space predefined by Δ*φ* and Δ*θ* (see Figure 3).
(5) In each subset *i* of a sector *i*, find the point *P*_max*i*_(*x*, *y*, *z*) with its maximum radius value (*r*_max*i*_) among all points in the subset.
(6) Calculate the volume of each pyramid with one vertex at the origin of the reference frame and four vertices at the points *P*_max_(*x*, *y*, *z*) of subsets belonging to four adjacent sectors (see Figure 4).
(7) Calculate the total volume of 3-D polyhedron (see Figure 5).

**Table 2:** Comparison of the total volumes of the accelerated polyhedrons in patients pre- and postsurgery.

		Presurgery		Postsurgery	
		EO	EC	EO	EC
FiS	Min (m^3^·s^−6^)	4.58·10^−4^	4.75·10^−4^	2.57·10^−4^	7.63·10^−4^
	Q1 (m^3^·s^−6^)	5.16·10^−4^	8.59·10^−4^	7.02·10^−4^	1.87·10^−3^
	Mdn (m^3^·s^−6^)	7.11·10^−4^	9.67·10^−4^	1.12·10^−3^	2.42·10^−3^
	Q3 (m^3^·s^−6^)	1.06·10^−3^	9.26·10^−3^	1.80·10^−3^	6.45·10^−3^
	Max (m^3^·s^−6^)	3.21·10^−3^	2.35·10^−2^	1.57·10^−2^	2.63·10^−2^

FoS	Min (m^3^·s^−6^)	2.31·10^−3^	4.70·10^−2^	2.31·10^−3^	2.73·10^−2^
	Q1 (m^3^·s^−6^)	4.22·10^−3^	5.48·10^−2^	5.89·10^−3^	2.06·10^−1^
	Mdn (m^3^·s^−6^)	1.04·10^−2^	1.45·10^−1^	7.00·10^−3^	5.78·10^−1^
	Q3 (m^3^·s^−6^)	1.18·10^−2^	5.22·10^−1^	3.67·10^−2^	1.24·10^+0^
	Max (m^3^·s^−6^)	3.25·10^−2^	2.40·10^+0^	4.49·10^−2^	8.95·10^+0^

EO: eyes open; EC: eyes closed; FiS: firm surface; FoS: foam surface; Min: minimum; Max: maximum; Mdn: median; Q1: first quartile; Q3: third quartile.

**Table 3:** Comparison of the total volumes of the polyhedrons of angles in patients pre- and postsurgery.

		Presurgery		Postsurgery	
		EO	EC	EO	EC
FiS	Min (deg^3^)	4.62·10^−2^	5.68·10^−2^	3.73·10^−2^	5.97·10^−2^
	Q1 (deg^3^)	9.12·10^−2^	1.30·10^−1^	7.62·10^−2^	2.61·10^−1^
	Mdn (deg^3^)	1.26·10^−1^	1.64·10^−1^	3.71·10^−1^	6.98·10^−1^
	Q3 (deg^3^)	2.18·10^−1^	9.26·10^−1^	7.99·10^−1^	3.60·10^+0^
	Max (deg^3^)	3.85·10^+0^	1.47·10^+1^	4.94·10^+0^	8.35·10^+0^

FoS	Min (deg^3^)	2.74·10^−1^	7.96·10^−1^	2.33·10^−1^	1.53·10^+0^
	Q1 (deg^3^)	3.42·10^−1^	1.21·10^+0^	2.73·10^−1^	7.61·10^+0^
	Mdn (deg^3^)	8.81·10^−1^	1.28·10^+1^	6.75·10^−1^	4.32·10^+1^
	Q3 (deg^3^)	1.68·10^+0^	1.83·10^+1^	3.28·10^+0^	7.41·10^+1^
	Max (deg^3^)	8.24·10^+0^	7.98·10^+1^	1.18·10^+2^	7.00·10^+2^

EO: eyes open; EC: eyes closed; FiS: firm surface; FoS: foam surface; Min: minimum; Max: maximum; Mdn: median; Q1: first quartile; Q3: third quartile.

**Table 4:** Comparison of the areas of the 95% confidence ellipses in patients pre- and postsurgery.

		Presurgery		Postsurgery	
		EO	EC	EO	EC
FiS	Min (mm^2^)	1.21·10^+2^	4.90·10^+1^	7.30·10^+1^	1.22·10^+2^
	Q1 (mm^2^)	1.74·10^+2^	1.88·10^+2^	1.48·10^+2^	4.45·10^+2^
	Mdn (mm^2^)	1.89·10^+2^	6.34·10^+2^	4.15·10^+2^	8.05·10^+2^
	Q3 (mm^2^)	4.66·10^+2^	1.04·10^+3^	6.60·10^+2^	1.10·10^+3^
	Max (mm^2^)	5.64·10^+3^	9.06·10^+3^	1.13·10^+3^	3.20·10^+3^

FoS	Min (mm^2^)	2.54·10^+2^	7.62·10^+2^	4.14·10^+2^	1.40·10^+3^
	Q1 (mm^2^)	5.05·10^+2^	1.08·10^+3^	5.49·10^+2^	4.79·10^+3^
	Mdn (mm^2^)	8.16·10^+2^	6.17·10^+3^	1.23·10^+3^	7.47·10^+3^
	Q3 (mm^2^)	1.59·10^+3^	8.49·10^+3^	1.60·10^+3^	1.16·10^+4^
	Max (mm^2^)	6.08·10^+3^	1.34·10^+4^	8.66·10^+3^	1.81·10^+4^

EO: eyes open; EC: eyes closed; FiS: firm surface; FoS: foam surface; Min: minimum; Max: maximum; Mdn: median; Q1: first quartile; Q3: third quartile.

**Table 5:** Comparison of the path lengths of CoP excursions in patients pre- and postsurgery.

		Presurgery		Postsurgery	
		EO	EC	EO	EC
FiS	Min (mm)	2.51·10^+2^	2.87·10^+2^	2.94·10^+2^	3.62·10^+2^
	Q1 (mm)	2.82·10^+2^	4.16·10^+2^	3.34·10^+2^	7.42·10^+2^
	Mdn (mm)	3.45·10^+2^	4.83·10^+2^	5.18·10^+2^	8.19·10^+2^
	Q3 (mm)	6.26·10^+2^	1.75·10^+3^	6.74·10^+2^	1.28·10^+3^
	Max (mm)	9.65·10^+2^	1.86·10^+3^	2.26·10^+3^	3.17·10^+3^

FoS	Min (mm)	5.44·10^+2^	1.61·10^+3^	3.62·10^+2^	1.94·10^+3^
	Q1 (mm)	6.62·10^+2^	1.82·10^+3^	7.42·10^+2^	2.96·10^+3^
	Mdn (mm)	1.00·10^+3^	3.63·10^+3^	8.19·10^+2^	4.17·10^+3^
	Q3 (mm)	1.45·10^+3^	4.33·10^+3^	1.28·10^+3^	4.44·10^+3^
	Max (mm)	2.05·10^+3^	5.35·10^+3^	3.17·10^+3^	6.94·10^+3^

EO: eyes open; EC: eyes closed; FiS: firm surface; FoS: foam surface; Min: minimum; Max: maximum; Mdn: median; Q1: first quartile; Q3: third quartile.

**Table 6:** The calculated *p* values from the Wilcoxon test to assess the differences between the results of stance trials.

		Presurgery	Postsurgery
TVPA	EO FiS versus EO FoS	<0.01^∗^	<0.01^∗^
	EC FiS versus EC FoS	<0.01^∗^	<0.01^∗^
	EO FiS versus EC FiS	0.05^∗^	<0.01^∗^
	EO FoS versus EC FoS	<0.01^∗^	<0.01^∗^

TVPD	EO FiS versus EO FoS	<0.01^∗^	0.07
	EC FiS versus EC FoS	<0.01^∗^	<0.01^∗^
	EO FiS versus EC FiS	0.12	<0.01^∗^
	EO FoS versus EC FoS	<0.01^∗^	<0.01^∗^

ACE	EO FiS versus EO FoS	<0.01^∗^	<0.01^∗^
	EC FiS versus EC FoS	<0.01^∗^	<0.01^∗^
	EO FiS versus EC FiS	0.07	0.03^∗^
	EO FoS versus EC FoS	<0.01^∗^	<0.01^∗^

PL	EO FiS versus EO FoS	<0.01^∗^	<0.01^∗^
	EC FiS versus EC FoS	<0.01^∗^	<0.01^∗^
	EO FiS versus EC FiS	<0.01^∗^	<0.01^∗^
	EO FoS versus EC FoS	<0.01^∗^	<0.01^∗^

TVPA: total volume of the polyhedron of accelerations; TVPD: total volume of the polyhedron of angles; ACE: area of the confidence ellipse; PL: path length of CoP; EO: eyes open; EC: eyes closed; FiS: firm surface; FoS: foam surface; ^∗^significant difference.

**Table 7:** Spearman's rank correlation coefficient between the volume of polyhedron and the area of the confidence ellipse of CoP excursions.

	Presurgery		Postsurgery	
	TVPA	TVPD	TVPA	TVPD
EO FiS	0.65^∗^	0.57^∗^	0.70^∗∗^	0.87^∗∗^
EO FoS	0.63^∗^	0.85^∗∗^	0.85^∗∗^	0.87^∗∗^
EC FiS	0.77^∗∗^	0.80^∗∗^	0.62^∗^	0.75^∗∗^
EC FoS	0.68^∗^	0.90^∗∗^	0.82^∗∗^	0.90^∗∗^

TVPA: total volume of the polyhedron of accelerations; TVPD: total volume of the polyhedron of angles; FiS: firm surface; FoS: foam surface; EO: eyes open; EC: eyes closed; ^∗^moderate correlation; ^∗∗^strong or very strong correlation.

**Table 8:** Spearman's rank correlation coefficient between the volume of polyhedron and the path length of CoP excursions.

	Presurgery		Postsurgery	
	TVPA	TVPD	TVPA	TVPD
EO FiS	0.90^∗∗^	0.67^∗^	0.78^∗∗^	0.92^∗∗^
EO FoS	0.82^∗∗^	0.97^∗∗^	0.51^∗^	0.60^∗^
EC FiS	0.91^∗∗^	0.90^∗∗^	0.53^∗^	0.73^∗∗^
EC FoS	0.95^∗∗^	0.87^∗∗^	0.90^∗∗^	0.72^∗∗^

TVPA: total volume of the polyhedron of accelerations; TVPD: total volume of the polyhedron of angles; FiS: firm surface; FoS: foam surface; EO: eyes open; EC: eyes closed; ^∗^moderate correlation; ^∗∗^strong or very strong correlation.

**Table 9:** The calculated *p* values from the Wilcoxon test to assess the differences between the results of measurements of patients pre- and postmicrosurgery.

TVPA	EO-FiS	0.13
	EO-FoS	0.50
	EC-FiS	0.91
	EC-FoS	0.10

TVPD	EO-FiS	0.30
	EO-FoS	0.91
	EC-FiS	0.50
	EC-FoS	0.02^∗^

ACE	EO-FiS	0.43
	EO-FoS	0.50
	EC-FiS	0.50
	EC-FoS	0.10

PL	EO-FiS	0.10
	EO-FoS	0.65
	EC-FiS	0.65
	EC-FoS	0.02^∗^

TVPA: total volume of polyhedron of the accelerations; TVPD: total volume of the polyhedron of angles; ACE: area of the confidence ellipse; PL: path length of CoP; FiS: firm surface; FoS: foam surface; EO: eyes open; EC: eyes closed; ^∗^significant difference.
